# Pelvic Ultrasound Findings in Women with Obstetric Fistula: A Cross-Sectional Study of Cases and Controls

**DOI:** 10.1155/2018/7409131

**Published:** 2018-01-10

**Authors:** Jeffrey P. Wilkinson, Angela M. Bengtson, Ennet Chipungu, Rachel J. Pope, Bonus Makanani, Margaret Moyo, Mwawi Mwale, Jennifer H. Tang

**Affiliations:** ^1^Baylor College of Medicine, Department of Obstetrics and Gynecology, Global Women's Health, Baylor Plaza, Houston, TX 77030, USA; ^2^University of North Carolina, Department of Epidemiology, 401 Rosenau Hall, Chapel Hill, NC 27599-7455, USA; ^3^Freedom from Fistula Foundation, Fistula Care Centre, Lilongwe, Malawi; ^4^Department of Obstetrics and Gynecology, Malawi College of Medicine, Blantyre, Malawi; ^5^Malawi Ministry of Health, Bwaila Maternity Hospital, Lilongwe, Malawi; ^6^University of North Carolina, Department of Obstetrics and Gynecology, 101 Manning Drive, Chapel Hill, NC 27599-7570, USA

## Abstract

**Objective:**

Obstetric fistula (OF) is a morbid condition caused by prolonged obstructed labor. Women with OF experience profound injury and have high rates of infertility and poor obstetric outcomes. We examined endovaginal ultrasound parameters in women with and without OF.

**Design/Setting/Sample/Methods:**

This cross-sectional study enrolled women evaluated at the Fistula Care Centre in Lilongwe, Malawi. Eligibility criteria included age 18–45, prior pregnancy, and a uterus on ultrasound. Participants underwent endovaginal ultrasound with measurement of cervical dimensions. Comparisons were done using *t*-tests and Fisher's exact test. Among women with OF, linear regression was used to assess whether fistula stage was associated with cervical length.

**Results:**

We enrolled 98 cases and 12 controls. Women with OF had shorter cervical lengths (18.8 mm versus 27.3 mm, *p* < 0.01), as well as shorter anterior (7.0 mm versus 9.3 mm, *p* < 0.01) and posterior (9.5 mm versus 11.0 mm, *p* < 0.04) cervical stroma, compared to controls.

**Conclusion:**

Women with OF have shorter cervical lengths and anterior and posterior cervical stroma, when compared to women without OF. This may offer a partial explanation for subfertility and poor obstetric outcomes in OF patients. Additional studies to clarify the role of ultrasound in OF patients and prediction of future fertility are warranted.

## 1. Introduction

Obstetric fistula (OF) is a devastating condition that occurs when women experience prolonged obstructed labor. The soft tissues of the pelvis undergo tissue necrosis, resulting in an abnormal communication between the vagina and the bladder and/or rectum. Women with OF continually leak urine and/or feces from their vagina until they are surgically repaired [1].

Many women with OF develop secondary infertility or amenorrhea, although some become pregnant again, before or after surgical repair. For those who do become pregnant, their pregnancies are often associated with high rates of preterm birth and spontaneous abortion, particularly in the second trimester [2–4]. The reasons for this are not well understood, although experts have hypothesized that women with OF have higher rates of preterm birth because of severe cervical damage and avulsion that occur during prolonged obstructed labor, which leads to cervical insufficiency [5]. In extreme cases, fistula surgeons have noted that patients have no identifiable cervical tissue [1, 6, 7]. A case series of 22 OF patients found that, on vaginal exam, 40% of patients had a normal cervix, 23% had cervical erosion, 18% had only a cervical dimple, 14% had a nonidentifiable cervix (because it was bound to the pubic symphysis), and 5% had a “harelip” defect of the anterior cervix. On transabdominal ultrasound, 68% had a normal-appearing cervix and 9% had anterior cervical defects [7]. No published study has assessed the use of endovaginal ultrasound to evaluate the cervix in OF patients.

Cervical length measurement is often performed during pregnancy to assess for short cervix, which is associated with preterm birth [8]. Women found to have a short cervix due to structural weakness of the cervix, as in the case of prior cone biopsy or a Müllerian duct anomaly, may benefit from placement of a prophylactic cerclage [9–11]. No published studies have examined the use of prepregnancy cervical length as a predictor for preterm birth in women at risk for having a short or abnormal cervix. The cervix lengthens during the first trimester of pregnancy, so pregnant women generally have longer cervices than nonpregnant women [12, 13]. The prepregnancy cervical length may have some predictive value, particularly in women with prior cervical trauma or surgery. For OF patients, pregnancy is uncommon, and they interface with the health care system infrequently. It is more practical to assess their pelvic anatomy by ultrasound at the time of their repair. If OF patients are found to have compromised pelvic anatomy, this could result in counseling on future pregnancy risk or potential interventions to prevent early pregnancy loss or other complications.

Therefore, we conducted this study to evaluate whether women with OF have a shorter prepregnancy cervical length than women without OF when measured by endovaginal ultrasound. We hypothesized that women with OF would have a significantly shorter cervical length when compared to women without OF.

## 2. Methods

This study was conducted at a 40-bed hospital unit dedicated to the care of obstetric fistula patients located on the grounds of the Bwaila Maternity Hospital in Lilongwe, Malawi. Between December 2012 and June 2014 (when funding for this study ended), a convenience sample of women who presented for surgical care of OF (defined as vesicovaginal fistula and/or rectovaginal fistula that developed during or immediately after labor) were recruited for participation as cases. Women who presented for evaluation of other gynecologic conditions (such as stress urinary incontinence or anal sphincter tears during childbirth) who did not have OF or a history of prolonged obstructed labor were recruited as controls. Inclusion criteria included women able to provide consent in Chichewa, aged 18–45 years, and a willingness to undergo the ultrasound examination at the time of surgery or during pelvic examination. Exclusion criteria included nulliparity, history of hysterectomy, current pregnancy, or presence of serious or terminal illness, such as advanced cervical cancer.

The study was approved by the Malawi National Health Sciences Research Committee, as well as the University of North Carolina Institutional Review Board/Office of Human Research Subjects. Prior to surgery in the cases or prior to examination in the controls, informed consent was obtained in writing after explanation by a native Chichewa speaker.

In study participants with OF, endovaginal ultrasound was performed after spinal anesthesia was administered for surgery. Most women had an empty bladder because of their OF, but in a few cases, there was sufficient urine to require catheterization prior to ultrasound. Endovaginal ultrasound was performed on all participants, by the same sonographer in lithotomy position. A systematic ultrasound examination was performed which measured the cervical length three times. The final cervical length was defined as the shortest and best of the three measurements. This was followed by measurement of cervical width, anterior and posterior cervical stroma, and uterine volume (length, width, and depth). Uterine position was assessed, and endometrial thickness, the percentage of the endometrium visualized, endometrial appearance and echogenicity, and other endometrial findings were noted. The presence or absence of fibroids including number, position, and size were also documented. Visualization of the ovaries was noted with ovarian volume measurements, presence of follicles, cysts, or other masses. Final uterine volume was calculated with the formula length × width × depth × 4/3 × π, and ovarian volume was calculated with the formula length × width × depth × π/6. The examiner noted his clinical impression of the likely cycle phase as part of the examination, based on the appearance of the endometrium and ovarian follicular development. The ovarian follicle data are published separately as part of an analysis of amenorrhea and hormone levels in OF patients [2].

Controls underwent endovaginal ultrasound in the lithotomy position during a pelvic examination after they had emptied their bladder. The same systematic approach to the ultrasound examination and measurements was performed.

Data were collected in a form designed for this study and then double entered and cleaned in a secure Research Electronic Data Capture (REDCap) study database [14], which is both web-based and password-protected. The REDCap database was managed by the North Carolina Translational and Clinical Sciences (NC TraCS) Institute and stored on a secure UNC server. The REDCap data were exported and then analyzed using STATA (StataCorp, College Station, TX).

## 3. Data Analysis

We compared demographic and clinical characteristics between cases with OF and controls without OF using Fisher's exact test. Cervical, endometrial, uterine, and ovarian characteristics were characterized for all women using descriptive statistics and compared between cases and controls using *t*-tests for continuous variables, the Mann-Whitney *U* test for ordinal variables, and Fisher's exact test for categorical variables. Among cases with OF who had a final cervical length measurement, we used linear regression to calculate mean differences and 95% confidence intervals in final cervical length by both major and minor Waaldijk classifications. All statistical analyses were done in Stata version 13 (StataCorp, College Station, Texas).

## 4. Results

A total of 110 women were included in the study: 98 cases with OF and 12 controls without OF. Both cases and controls were most likely to be 25–34 years of age, to have given birth to 2-3 children, be HIV-uninfected, and have a normal body mass index (18.5–25.0). While not statistically different, women with OF were more likely to not be married (32% versus 8%), to have not gone to school or only have gone to primary school (93% versus 75%), and to be a peasant farmer (71% versus 58%), compared to women without OF (Table 1).

The cervix could be visualized and measured in 90 of the 98 women with OF and all women without OF. Women with OF were found to have significantly shorter cervical lengths than women without OF, after adjustment for parity (18.8 mm versus 27.3 mm, *p* value < 0.01). Both anterior and posterior cervical stroma were shorter in women with OF than controls (7.0 mm versus 9.3 mm, *p* value 0.01, and 9.5 mm versus 11 mm, *p* value 0.04, resp.). There were no significant differences in cervical width, endometrial thickness, or uterine volume between cases and controls. The endometrium was fully visualized in 78% of cases and 100% of controls (*p* value 0.07). No significant differences were noted in other endometrial or ovarian characteristics (Table 2).

Of the 98 women included with obstetric fistula, 90 had a final cervical length measured (92%). Overall, the largest proportion of women had a fistula categorized as Waaldijk Major IIAb/IIBb (*n* = 40, 44%) or Waaldijk Minor large/extensive (*n* = 30, 33%). Women with more severe fistula classifications had slightly shorter final cervical lengths on average. However, there were no statistically significant associations between stage of the fistula as determined by the Waaldijk classification system and cervical length (Table 3).

## 5. Discussion

This cross-sectional endovaginal ultrasound study compared women with OF caused by prolonged obstructed labor to women without OF or prolonged obstructed labor. Women with OF had significantly shorter cervical lengths and anterior cervical stroma. This is the first study to systematically examine the endovaginal ultrasound characteristics of the cervix and other pelvic organs in women with OF. Often, one is unable to visualize or palpate the cervix in OF patients as the cervix is embedded in scar or absent altogether as a result of pressure necrosis. However, even in patients with apparent absence of the cervix by direct visualization, one can ultrasonographically visualize residual cervical tissue. The extent to which this cervix is functionally useful for future fertility and parturition remains uncertain.

Of important note is that we could not visualize the cervix in 8 of the 98 OF patients while we were able to visualize the uterus in all patients. The most likely explanation for nonvisualization of the cervix is that no cervix was present (i.e., the cervix was completely destroyed by the obstructed labor). In this case, their cervical length measurements would be 0 mm, thereby strengthening our findings of shorter cervical lengths in OF patients. If all OF patients with a cervix that could not be visualized were considered to have cervical length of 0 mm, the average cervical length among OF patients would drop from 18.8 mm to 17.2 mm. However, we cannot rule out nonvisualization secondary to sonographer error or the limitations of our ultrasound equipment.

This study has a few additional potential limitations. First, the physician who performed the ultrasound examination and surgery was not blinded to the fistula status of the patients. The study design would have prohibited blinding in that ultrasound in OF was performed in the operating theatre. Compromised vaginal anatomy including frequent vaginal stenosis and severe scarring often makes a comprehensive examination impossible prior to anesthesia in OF patients. Also, independent of the site of examination, it would be impossible to blind an examiner to the status of a fistula patient. Another limitation was that the controls represented far fewer patients than the cases. Unfortunately, in this study, it was unavoidable. The fistula center where the study was performed primarily cares for women with OF; hence, there were limited opportunities to recruit women without an OF or a history of prolonged obstructed labor (controls). Despite the highly statistically significant differences we found between cases and controls, the limited number of controls could have resulted in an unrepresentative sample due to unexpected deviants or selection bias related to their presentation to the fistula center for care. We are reassured that the average cervical length in controls was 27.3 mm which is more consistent if not somewhat shorter than that found in prior studies which measured cervical length in the nonpregnant patient [12, 13]. Indeed, if there was systematic error in measuring the cervical length towards the lower range, this would have resulted in less statistically significant findings, so this issue is unlikely to have affected our results. Unfortunately, there are no published data for average cervical length in our study population to which we can compare our results.

Despite these limitations, the strengths of this study prevail. There is clear pathophysiologic plausibility to the findings of cervical destruction during the process of obstructed labor. Surgically, we commonly find complete destruction of the bladder base, trigone, anterior vagina, and cervix. It is common to have difficulty finding the cervical os during surgery for OF, or when the cervix is found, there is often no identifiable anterior cervical stroma. A common surgical challenge when repairing these patients is prevention of cervical stenosis and subsequent amenorrhea and hematometra after surgery.

## 6. Conclusion

The implications for the findings of this study are numerous. Up to 70% of women who experience OF will never have a living child [15]. Determining the role of cervical destruction in the etiology of subsequent infertility, subfertility, or pregnancy loss is an important area of future exploration. There may be opportunities to study the impact of surgical revision of the cervix at the time of OF surgery. Also, OF patients who are found to have a shortened cervix may benefit from cerclage, pessary, hormonal therapy, or other modalities to prevent preterm delivery or early pregnancy loss; however, this would have to be validated with an appropriate prospective study before implementation.

In the rush to cure women and girls with OF from their debilitating urinary incontinence, we have largely neglected other important aspects of women's health care in this vulnerable population. Fecundity and the ability to carry a pregnancy to viability are often as important to our patients as achieving continence. Understanding the role of compromised pelvic anatomy infertility in this population is a critical step to helping these patients realize their reproductive goals.

## Figures and Tables

**Table 1:** Demographic and clinical characteristics of 110 Malawian women seeking care at an obstetric fistula center in Lilongwe, Malawi, between December 2012 and June 2014.

Variable	Obstetric fistula (*N* = 98)	No obstetric fistula (*n* = 12)	*p* value^a^
	*N* (%)	*N* (%)	
Age			0.57
18–24	21 (21.4)	2 (16.7)	—
25–34	47 (48.0)	8 (66.7)	—
35–45	30 (30.6)	2 (16.7)	—
Parity			0.37
1	27 (27.6)	1 (8.3)	—
2-3	38 (38.8)	6 (50.0)	—
≥4	33 (33.7)	5 (41.7)	—
BMI^b^			0.81
<18.5	8 (9.0)	2 (18.2)	—
18.5–<25.0	54 (71.9)	8 (72.7)	—
25.0–<30.0	11 (12.4)	1 (9.1)	—
≥30	6 (6.7)	0 (0.0)	—
Marital status			0.17
Not married	31 (31.6)	1 (8.3)	—
Married	67 (68.4)	11 (91.7)	—
Education			0.08
None or primary	91 (92.9)	9 (75.0)	—
Secondary or more	7 (7.1)	3 (25.0)	—
Occupation			0.34
Other^c^	28 (28.6)	5 (41.7)	—
Peasant farmer	70 (71.4)	7 (58.3)	—
HIV status			0.24
Positive	5 (5.1)	2 (16.7)	—
Negative	84 (85.7)	9 (75.0)	—
Don't know	9 (9.2)	1 (8.3)	—

^a^Fisher's exact test; ^b^BMI: body mass index, calculated as kg/m^2^; ^c^“Other”:  unemployed, housewife or caretaker, large or small business owner, casual laborer, other, don't know. Missing data: BMI *n* = 10.

**Table 2:** Differences in obstetric characteristics by obstetric fistula status, among 110 women in Malawi.

Variable	Obstetric fistula (*N* = 98)	No obstetric fistula (*n* = 12)	*p* value	Missing
	Mean (SD) or *N* (%)	Mean (SD) or *N* (%)		
Final cervical length, mm	18.8 (8.7)	27.3 (6.2)	<0.01	8
Cervical width, mm	18.5 (8.0)	21.45 (3.0)	0.21	8
Anterior cervical stroma, mm	7.0 (3.1)	9.3 (1.4)	0.01	10
Posterior cervical stroma, mm	9.5 (2.5)	11.0 (1.5)	0.04	9
Final uterine volume, cm^3^	587.7 (279.8)	628.2 (303.3)	0.64	0
Endometrial thickness, mm	7.0 (5.1)	7.1 (5.2)	0.96	0
Endometrium visualized			0.07	0
100%	76 (77.6)	12 (100.0)	—	—
51-99%	12 (12.2)	0 (0.0)	—	—
≤50%	9 (9.2)	0 (0.0)	—	—
0%	1 (1.0)	0 (0.0)	—	—
Endometrial appearance			0.81	0
Homogeneous	67 (68.4)	8 (66.7)	—	—
Heterogeneous	20 (20.4)	2 (16.7)	—	—
Trilaminar	10 (10.2)	2 (16.7)	—	—
Not seen	1 (1.0)	0 (0.0)	—	—
Endometrial echogenicity				0
Hyperechoic	61 (62.2)	10 (83.3)	0.48	—
Isoechoic	25 (25.5)	1 (8.3)	—	—
Hypoechoic	11 (11.2)	1 (8.3)	—	—
Not seen	1 (1.0)	0 (0.0)	—	—
Other endometrial findings				
Fluid filled	25 (25.5)	2 (16.7)	0.73	—
Cystic	0 (0.0)	0 (0.0)	—	—
Serpiginous	6 (6.1)	0 (0.0)	0.49	—
Other	1 (1.0)	0 (0.0)	0.89	—
Not assessed	36 (36.7)	4 (33.3)	0.54	—
Not seen	31 (31.6)	6 (50.0)	0.17	—
Not sure	0 (0.0)	0 (0.0)	—	—
Final right ovarian volume, ml	11.4 (9.7)	9.9 (3.2)	0.65	28
Final left ovarian volume, ml	11.0 (10.8)	12.0 (4.0)	0.76	22
Total antral follicle count	14.4 (6.4)	12.3 (5.3)	0.40	44
Impression of cycle phase			1.00	0
Follicular	59 (60.2)	8 (66.7)	—	—
Periovulatory	23 (23.5)	3 (25.0)	—	—
Luteal	15 (15.3)	1 (8.3)	—	—
Not sure	1 (1.0)	0 (0.0)	—	—

**Table 3:** Bivariable associations between Waaldijk classification and final cervical length among 90^∗^ Malawian women with obstetric fistula.

Classification	*N*	Mean (SD)	Mean difference (95% CI)	*p* value
Waaldijk major				
I	28	20.0 (8.0)	0.00	—
IIAa	15	18.3 (6.3)	−1.70 (−7.45, 4.05)	0.56
IIAb/IIBb	40	18.1 (10.4)	−1.99 (−6.42, 2.44)	0.38
*Missing*	*7*	—	—	—
Waaldijk minor				
Small	26	19.8 (6.1)	0.00	—
Medium	26	19.6 (10.1)	−0.19 (−5.14, 4.76)	0.94
Large/extensive	30	16.8 (9.9)	−2.97 (−7.76, 1.81)	0.22
*Missing*	*8*	—	—	—

^∗^8 women with obstetric fistula excluded due to missing final cervical length measurement.
